# Comparative evaluation of human ß defensin-2 in GCF and microbial flora in subgingival plaque among patients treated with conventional orthodontic braces and aligners: a prospective clinical study

**DOI:** 10.1590/2177-6709.30.6.e252541.oar

**Published:** 2026-01-23

**Authors:** Aditi SHARMA, Mukesh KUMAR, Ekta YADAV, Sumit KUMAR, Manish GOYAL, Sonal RASTOGI

**Affiliations:** 1 Teerthanker Mahaveer University, Teerthanker Mahaveer Dental College and Research Centre, Department of Orthodontics and Dentofacial Orthopaedics (Moradabad, Uttar Pradesh, India). Teerthanker Mahaveer University Teerthanker Mahaveer Dental College and Research Centre Department of Orthodontics and Dentofacial Orthopaedics Moradabad Uttar Pradesh India

**Keywords:** Comparative, Human, Defensin-2, GCF, Estudo comparativo, Humanos, Defensina-2, FCG

## Abstract

**Objective:**

The aim of this study was to compare the presence of human ß defensin-2 (hBD-2) in GCF and microbial flora in the subgingival plaque, among the patients treated with fixed orthodontic braces and aligners.

**Methods:**

Samples of GCF and subgingival plaque were collected from 170 patients (85 conventional metal fixed appliance and 85 aligner patients) at T0 (baseline), T1 (14 days), T2 (1 month) and T3 (3 months). The GCF sample was analyzed for the presence of hBD-2 using ELISA. The subgingival plaque sample was analyzed for the presence of *Porphyromonas gingivalis*, *Prevotella intermedia*, *Actinobacillus actinomycetemcomitans*, and *Treponema denticola* using RT-PCR. For statistical analysis, intergroup comparison was done using independent z-test, to compare the presence of hBD-2, as well as the microbial flora. Repeated measure ANOVA with *post-hoc* analysis was done to evaluate hBD-2 and subgingival plaque at different time periods.

**Results:**

On intergroup comparison, the presence of hBD-2 was found to be significantly (p≤0.05) greater at T2 and T3 in the conventional than in the aligner group. The presence of the aforementioned bacteria was found to be significantly (p≤ 0.05) greater at T1, T2 and T3 in the conventional braces group than in the aligner group. On intragroup evaluation, for both the parameters, the minimum value was seen at T0 and the maximum, at T3 for the conventional and aligner groups.

**Conclusion:**

The levels of hBD-2 and subgingival bacteria was found to be greater in the conventional group than the aligner group.

## RESUMO

**Objetivo:**

O objetivo deste estudo foi comparar a presença da ß-defensina-2 humana (hBD-2) no fluido crevicular gengival (FCG) e a flora microbiana na placa subgengival em pacientes tratados com aparelhos ortodônticos fixos e alinhadores.

**Métodos:**

Amostras de FCG e placa subgengival foram coletadas de 170 pacientes (85 com aparelho fixo metálico convencional e 85 com alinhadores) nos tempos T0 (inicial), T1 (14 dias), T2 (1 mês) e T3 (3 meses). A amostra de FCG foi analisada para a presença de hBD-2 por meio de ELISA. A amostra de placa subgengival foi analisada para a presença de *Porphyromonas gingivalis, Prevotella intermedia, Actinobacillus actinomycetemcomitans* e *Treponema denticola* utilizando RT-PCR. Para análise estatística, a comparação entre os grupos foi realizada utilizando o teste z independente, para comparar a presença de hBD-2, bem como a flora microbiana. ANOVA de medidas repetidas com análise *post-hoc* foi realizada para avaliar a hBD-2 e a placa subgengival em diferentes períodos de tempo.

**Resultados:**

Na comparação entre os grupos, a presença de hBD-2 foi significativamente maior (p≤0,05) em T2 e T3 no grupo com aparelho convencional do que no grupo com alinhadores. A presença das bactérias mencionadas anteriormente foi significativamente maior (p≤0,05) em T1, T2 e T3 no grupo com aparelho convencional do que no grupo com alinhadores. Na avaliação intragrupo, para ambos os parâmetros, o valor mínimo foi observado em T0 e o máximo, em T3, tanto para o grupo com aparelho convencional quanto para o grupo com alinhadores.

**Conclusão:**

Os níveis de hBD-2 e de bactérias subgengivais foram maiores no grupo convencional do que no grupo com alinhadores.

## INTRODUCTION

Orthodontic tooth movement (OTM) occurs due to a combination of bone formation and bone resorption. This course of events occurs in response to the inflammatory reaction generated due to prolonged period of force administration, and is associated with the production and release of certain biomarkers.^1^^,^^2^ These biomarkers, evident during OTM, may be segregated from gingival crevicular fluid (GCF).

The GCF is an inflammatory exudate found in the sulcus of human gingiva, first defined by Alfano.^3^ The GCF flow increases during inflammation, and its composition becomes similar to that of inflammatory exudate. High rate of GCF flow flushes the bacterial colonies, along with their metabolites, away from the sulcus, aiding in host defense. GCF diffuses primarily through the basement membrane, the junctional epithelium and, finally, into the sulcus.^4^ One of such biomarkers is human ß defensin- 2 (hBD-2). The defensins are one of the antimicrobial peptides having a high-level microbicidal effect against a wide variety of microorganisms. These peptides help enhance the acquired immunological response.^5^ hBD-1, hBD-2 and hBD-3 are the three types of human ß defensins that have been found in the oral cavity. hBD-2 production has been found to escalate during the occurrence of infection or inflammation caused by the presence of periodontal pathogens such as *P. gingivalis*.^6^^,^^7^

OTM may also have an influence on the general microbiota of the oral cavity. It may result in an increase in the accumulation of subgingival plaque, thus influencing the general equilibrium of microflora in mouth.^8^ Fixed orthodontic appliances have been shown to promote the growth of a subgingival plaque containing periodontopathogenic bacterial strains such as *Porphyromonas gingivalis* (*P. gingivalis*), *Prevotella intermedia* (*P. intermedia*), *Actinobacillus actinomycetemcomitans* (*A. actinomycetemcomitans), Fusobacterium nucleatum (F. nucleatum)* and *Treponema denticola (T. denticola)*. On the other hand, removable orthodontic appliances enable good oral hygiene, lowering the risk of occurrence of detrimental dental and periodontal complications.^9^

Many studies have been done evaluating the levels of biomarkers such cytokines in aligners, as well as fixed orthodontic appliances. Also, studies for the evaluation of subgingival plaque during orthodontic treatment have been done. However, both these studies have been done individually, and no study has been done evaluating as well as comparing the release of human ß defensin- 2 during OTM and the presence of subgingival microbial flora during orthodontic treatment. Hence, the aim of this study was to compare the presence of human ß defensin- 2 (hBD-2) and subgingival plaque among the patients with fixed orthodontic appliance and clear aligner patients.

## MATERIAL AND METHODS

This clinical study was carried out in the Department of Orthodontics and Dentofacial Orthopaedics after approval by the Institutional Ethical Committee (TMDCRC IEC/21-22/OD02). A sample size^10^ (power = 90%) of 170 was estimated, which included 85 patients per group. The first group was conventional metal fixed appliance patients, and the second group was aligner patients.

An information sheet explaining the details of the study was provided to the participants, and a signed written informed consent form was obtained from them.

The inclusion criteria were:

1. Patients willing for fixed orthodontic treatment.
2. Patients with Class I malocclusion.
3. Patients willing for aligner treatment.
4. Non extraction cases.
5. Patients with moderate to severe crowding in the lower anterior segment according to Little’s irregularity index.^11^
6. Age group: young adult group (18-25 years)^12^

The exclusion criteria were:

1. Patients with Class II or Class III malocclusion.
2. Medically compromised patients.
3. Patients on any systemic medication.
4. Periodontally compromised patients.
5. Patients with history of any orthodontic treatment.
6. Patients with deleterious habits.

In patients of conventional metal fixed appliance group, bonding of both the upper and lower arches was done using pre-adjusted Edgewise appliance, 0.022 x 0.018-in slot, MBT prescription brackets, and 0.009-in ligature wire was used to ligate the brackets. For the aligner patients’ group, ODONTO Clear Aligners (ODONTO Aligners, Mumbai, India) were used. All patients were advised to change the aligner every 15 days, in accordance with the individual treatment plan that was pre-decided for each patient. 

All the patients underwent oral prophylaxis two weeks before the beginning of the study.^10^ The patients were asked to use an orthodontic toothbrush and dental floss during the duration of the treatment.^9^ Plaque and gingival indices were used to assess the oral health of the patient initially, and patients with score zero were included in the study.^13^

After selection of the groups, according to the aforementioned criteria, GCF sample (0.5-1 ul) and subgingival plaque sample was collected from the patients in both groups. Before sample collection, the area was completely washed with water, the tooth was isolated with cotton rolls and the area, dried, to stop salivary contamination.^14^ The sample collection was done at the following time periods^2^: T0 (baseline), T1 (14 days), T2 (1 month) and T3 (3 months). The sample collection and examination were performed by the same individual.

The chosen site of collection for the study in both the samples was the most crowded teeth in the lower anterior segment, which was determined according to the Little’s irregularity index.^11^

For the GCF sample collection, microcapillary tubes were used. Any sample contaminated by saliva or blood was discarded. The collected samples were promptly transferred in 0.5ml Eppendorf tubes containing buffered-phosphate saline with a pH of 7.0. Centrifugation was done at 13,000 g at 48°C for 15 minutes. Following it, the samples were stored at -80°C until analysis.^15^ The GCF samples were tested for the presence of the inflammatory biomarker human ß defensin- 2, using enzyme-linked immunosorbent assay (ELISA). The result was then expressed in pg/ul.

For the subgingival plaque, the sample was collected using a subgingival curette. It was then placed in vials containing Tris-EDTA (TE) buffer. The vials were centrifuged at 5000 rpm, for five minutes. Following it, DNA was extracted and stored at -20°C for further procedure of real time polymerase chain reaction (RT-PCR)^16^. The subgingival plaque was tested for the presence of *P. gingivalis, P. intermedia, A. actinomycetemcomitans,* and *T. denticola.* The quantity obtained was as number of bacterial cells per reaction.

## STATISTICAL ANALYSIS

Descriptive and inferential statistics were analyzed using IBM SPSS version 20.0 (IBM Corp. Released 2011. IBM SPSS Statistics for Windows, Version 20.0. Armonk, NY: IBM Corp). The Shapiro-Wilk test was used to investigate the distribution of the data, and Levene’s test was used to explore the homogeneity of the variables. The data were found to be homogeneous and normally distributed. Mean and standard deviation (SD) were computed for each variable.

Independent sample z-test was used to compare the release of human ß defensin- 2 in GCF and comparison of the plaque count of bacteria among the patients with aligners and fixed orthodontics. Repeated measure ANOVA with *post-hoc* analysis was done for the intragroup comparison of release of human ß defensin- 2 in GCF, and comparison of the plaque count of bacteria among the patients using aligners and fixed orthodontics, at different time intervals. A p≤0.05 was considered as statistically significant difference.

## RESULT

### INTERGROUP COMPARISON OF THE PRESENCE OF HUMAN β DEFENSIN-2 IN GCF

For hBD-2, significant difference was observed between aligner and conventional groups at T2 (1 month) and T3 (3 months) (p≤0.05) (Table 1). The concentration for hBD-2 was higher in the conventional, as compared to the aligner group at every stage. For the conventional group, the concentrations were 10.55±1.61 at T1, 17.50±1.95 at T2, and 48.65±4.62 at T3, while for aligner, 9.85±1.37 at T1, 13.64±2.38 at T2, and 21.22±1.41 at T3.

**Table 1:** Intergroup comparison of human β defensin- 2 in GCF.

		Mean	SD	P value
T0	Conventional	5.0556	1.23906	0.211
	Aligners	4.4444	1.57092	
T1	Conventional	10.5556	1.61021	0.336
	Aligners	9.8556	1.37214	
T2	Conventional	17.5000	1.95256	0.001*
	Aligners	13.6444	2.38281	
T3	Conventional	48.6556	4.62361	0.001*
	Aligners	21.2222	1.41136	

Independent z test with (p≤0.05) is significant. *p≤0.05.

### INTERGROUP COMPARISON OF THE MICROBIAL LEVELS IN SUBGINGIVAL PLAQUE

For microbial levels in subgingival plaque, a significant difference (p<0.05) was observed between the conventional and the aligner group at T1 (14 days), T2 (1 month) and T3 (3 months) for all the bacteria, i.e., *A. actinomycetemcomitans, P. gingivalis, T. denticola* and *P. intermedia* (Table 2). The highest value for *A. actinomycetemcomitans* was seen in the conventional group at T3 (37585.55±18844.54) and lowest was seen for the aligner group at T0 (586.67±892.28).

**Table 2:** Intergroup comparison of subgingival plaque levels of microbes levels.

	Time	Group	Mean	SD	P-**value**
*A. actinomycetemcomitans*	T0	Conventional	6490.22	10407.15	0.301
		Aligners	5861.67	892.24	
	T1	Conventional	13182.22	17016.12	0.001*
		Aligners	1477.83	1800.93	
	T2	Conventional	23356.66	19831.24	0.001*
		Aligners	2664.43	2330.76	
	T3	Conventional	37585.55	18844.54	0.001*
		Aligners	3861.13	2650.97	
*P. gingivalis*	T0	Conventional	1978.88	16497.65	0.231
		Aligners	1846.67	911.37	
	T1	Conventional	28315.55	21990.29	0.001*
		Aligners	2678.93	1134.66	
	T2	Conventional	47365.55	21200.74	0.001*
		Aligners	4655.63	644.92	
	T3	Conventional	70870.00	18971.99	0.001*
		Aligners	6300.03	854.03	
*T. denticola*	T0	Conventional	1163.33	1545.70	0.111
		Aligners	1038.93	1115.08	
	T1	Conventional	3409.44	1599.60	0.001*
		Aligners	3633.33	589.06	
	T2	Conventional	5694.44	2800.93	0.001*
		Aligners	240.03	654.77	
	T3	Conventional	29677.77	15806.11	0.001*
		Aligners	6701.13	1011.76	
*P. intermedia*	T0	Conventional	1348.88	15347.86	0.000
		Aligners	1310.00	615.14	
	T1	Conventional	29043.33	25564.02	0.001*
		Aligners	1530.03	1557.05	
	T2	Conventional	48188.88	31754.47	0.001*
		Aligners	2975.63	1868.10	
	T3	Conventional	247722.22	149478.62	0.001*
		Aligners	3703.33	2260.20	

Independent z test with (p≤0.05) is significant. *p≤0.05.

Similarly, for *P. gingivalis*, the highest concentration was at T3 (70870.00±18971.99) in the conventional group, and the smallest concentration was at T0 (846.67±911.37) in the aligner group. It was also observed that for *T. denticola*: the minimum concentration was for aligner at T0 (1038.93±1115.08), and the maximum concentration was at T3 (29677.77±15806.11) for the conventional group. For *P. intermedia*, the value for conventional group at T3 was 247722.22±149478.62, which was the maximum concentration observed for the aforementioned bacteria and the value for aligner group at T0 was 1310.00±615.14, which was the minimum concentration recorded for the same.

### INTRAGROUP COMPARISON OF THE PRESENCE OF HUMAN β DEFENSIN- 2 IN GCF

It was observed that there was a significant difference (p<0.05) among the levels of hBD-2 at different time intervals (Table 3). The values for conventional group was 5.056±1.23 at T0, 10.556±1.61 at T1, 17.500±1.95 at T2, and 48.655±4.23 at T3, whereas for aligners, it was 4.44±1.57 at T0, 9.85±1.37 at T1, 13.64±2.38 at T2, and 21.22±1.41 at T3.

**Table 3:** Intragroup comparison of human β defensin- 2 in GCF.

	T0	T1	T2	T3	P value
Conventional	5.05±1.23^a^	10.556±1.610^b^	17.500±1.952^c^	48.655±4.236^d^	0.001*
Aligners	4.44±1.57^a^	9.85±1.37^b^	13.64±2.38^c^	21.22±1.41^d^	0.001*

Repeated Measures ANOVA with p≤0.05 is significant. Different letters in the same row represent statistically significant differences among different groups. *p≤0.05.

### INTRAGROUP COMPARISON OF LEVELS OF MICROBES FOR THE CONVENTIONAL GROUP AND ALIGNER GROUP

Intragroup comparison of levels of microbes (*A. actinomycetemcomitans, P. gingivalis, T. denticola* and *P. intermedia*) for conventional group (Table 4) as well as aligner group (Table 5) showed a significant difference (p≤0.05) at every time interval. For all the bacteria, the minimum value was found at T0 and the maximum value was found at T3 in both the groups. For the conventional group, the values at T0 for the aforementioned bacteria were 6490.22±10407.15, 1978.88±16497.65, 1163.33±1545.70 and 1348.88±15347.86 respectively, and the values at T3 were 37585.555±18844.54, 70870.0±18971.99, 29677.77±15806.11 and 247722.22±149478.6 respectively. For the aligner group, the values at T0 were 5861.67±892.28, 1846.67±911.37, 1038.93±1115.08, and 1310.00±615.14, respectively; and the values at T3 were 3861.13±2690.97, 6300.03±854.03, 6701.13±1011.76, and 3703.33±2260.20, respectively.

**Table 4:** Intragroup comparison of subgingival plaque levels of microbes in conventional patients.

	T0	T1	T2	T3	p-value
*A. actinomycetemcomitans*	6490.22± 10407.15^a^	13182.222± 17016.12^b^	23356.66± 19833.203^c^	37585.555± 18844.54^d^	0.001*
*P. gingivalis*	1978.88± 16497.65^a^	28325.555± 21990.28^b^	47365.555± 21700.73^c^	70870.0± 18971.99^d^	0.001*
*T. denticola*	1163.33± 1545.70^a^	3409.44± 1599.60^b^	5694.44± 2800.93^c^	29677.77± 15806.11^d^	0.001*
*P. intermedia*	1348.88± 15347.86^a^	26043.33± 25564.02^b^	48188.88± 31754.46^c^	247722.22± 149478.6^d^	0.001*

Repeated measures ANOVA with p≤0.05 is significant. Different letters in the same row represent statistically significant differences among different groups. *p≤0.05.

**Table 5:** Intragroup comparison of subgingival plaque levels of microbes in aligner patients.

	T0	T1	T2	T3	p-value
*A. actinomycetemcomitans*	5861.67± 892.28^a^	1477.83± 1800.93^b^	2664.43± 2330.76^c^	3861.13± 2690.97^d^	0.001*
*P. gingivalis*	1846.67± 911.37^a^	2678.93± 1134.66^b^	4655.63± 644.92^c^	6300.03± 854.03^d^	0.001*
*T. denticola*	1038.93± 1115.08^a^	3633.33± 589.06^b^	5240.03± 654.77^c^	6701.13± 1011.76^d^	0.001*
*P. intermedia*	1310.00± 615.14^a^	1530.03± 1557.05^b^	2975.63± 1868.11^c^	3703.33± 2260.20^d^	0.001*

Repeated measures ANOVA with p≤0.05 is significant. Different letters in the same row represent statistically significant differences among different groups. *p≤0.05.

## DISCUSSION

This study analyzed the level of hBD-2 in GCF and the level of microbial flora in subgingival plaque among two groups of patients undergoing orthodontic treatment, one with fixed appliances and the other, using aligners.

Orthodontic tooth movement (OTM) is characterized by the creation of compression and tension zones in periodontal ligaments (PDL), leading to the remodelling of bone.^17^ Orthodontic treatment also leads to the induction of physiologically active substances inside the periodontium, which ultimately causes cellular response in various microenvironments for biological response.^18^ Biomarkers are released as feedback to this cellular mechanism.

Biomarkers are indicators of normal/pathologic biological processes.^1^ They can be measured and assessed objectively.^18^ Due to its sensitivity and specificity, biomarkers are able to provide information about the biological situation, such as the alterations in the periodontal tissue, in certain stages of OTM.^19^

The GCF is highly useful for testing the presence of biomarkers. The GCF is an assemblage of elements coming from host inflammatory cells, periodontium structural cells, serum, and oral microorganisms.^20^ It is present in the gingival sulcus. The gingival sulcus is continuous with the PDL, and can be conveniently accessed in the oral cavity. Although the PDL could serve as a more effective site for measuring the changes related to biomarkers, it is difficult to acquire the sample needed directly from it. Hence, the GCF was chosen in this study for collection of the required sample. Many previous studies have reported successfully using the GCF sample from the gingival sulcus for the detection of biochemical markers.^1^^,^^2^^,^^9^^,^^10^^,^^21^

Defensins trigger the production of proinflammatory cytokines by the process of chemoattraction of macrophages, monocytes, mast cells and T-lymphocytes. Antigen specific immune responses have been shown to be enhanced by human ß-defensins, which in turn suppresses the production of the proinflammatory cytokines.^22^ Human ß defensins-2 is a 41 amino acid antimicrobial peptide rich in cysteine, which was first discovered in human skin in 1997.^23^ It has been shown to be significantly found during the course of any inflammatory process occurring due to infection such as gingivitis. It has also been suggested in previous studies that OTM may lead to gingivitis, which results an increase in the release of Human ß defensins-2. Also, many studies conducted have reported that OTM may cause some adverse effect on the periodontium.^24^

A study by Gujar et al.^10^ compared the cytokine levels in GCF during orthodontic treatment with aligners and conventional labial fixed appliance. It was reported that the cytokine levels were increased in patients who were treated with conventional fixed labial appliance, when compared to patients who underwent treatment with aligners. However, no study could be found in the literature evaluating and comparing the level of hBD-2 in patients with aligner and conventional brackets. This study revealed that the level of hBD-2 was found to be higher in patients of conventional orthodontic brackets as compared to the aligner patients.

Plaque accumulation may alleviate gingival inflammation, leading to increased bacterial accumulation.^9^ In an investigation by Levrini et al.^9^, comparing the periodontal health status in patients treated with aligner and fixed orthodontics, it was revealed that the patients who underwent treatment with aligners had an overall better periodontal health. The result of the present study was in accordance with the study of Lombardo et al.^25^, who reported that the total bacterial load present in the oral cavity increased in fixed appliance group, as compared to clear aligner group.

In this study, the quantity of *P. gingivalis, P. intermedia, A. actinomycetemcomitans,* and *T. denticola* was checked because these are most commonly seen in orthodontic patients. Various studies identified the strongest disease link between *A. actinomycetemcomitans, P. gingivalis,* and *Prevotella intermedia* in orthodontic patients.^26^^,^^27^ Subgingival colonization of *A. actinomycetemcomitans* was found in this study, which is similar to the result of the study by Paolantonio et al.^28^, who reported that, in patients undergoing orthodontic treatment with fixed appliance, increased growth of the bacteria was observed at the placement site of orthodontic bands and brackets. In a systematic review by Papageorgiou et al.^29^, it was observed that the level of *A. actinomycetemcomitans* was found to be elevated in patients with fixed orthodontic appliances.

Naranjo et al.^30^, in their study, also evaluated the presence of *P. intermedia* and *P. gingivalis* in patients with brackets, and found a significant increase in the levels of these bacteria. This was similar to the result of this study. Gong et al.^31^ demonstrated that patients undergoing fixed orthodontic therapy had remarkable levels of *A. actinomycetemcomitans, P. gingivalis, P. intermedia* and *T. denticola,* which resulted in gingival enlargement and, hence, they may be capable of releasing inflammatory biomarkers, which might influence the inflammatory and immune responses. The result of this study showed that a higher amount of microbial flora was present in patients undergoing orthodontic treatment with fixed appliances, in comparison to aligner patients.

Intragroup comparison of levels of hBD-2 at different time periods showed that there was an increase in its level from T0 (baseline) to T3 (months) in both the groups. However, it also became evident that the increase was greater in the conventional treatment group than in the aligner group. For the subgingival bacterial group, it was again observed that the bacterial count was minimum at T0 and maximum at T3 for each of the bacteria. This was similar to the study results of Naranjo et al.^30^, who found that higher count of microorganisms was found after three months of treatment. A study by Levrini et al.^9^ also concluded that there was a substantial increase in the total biofilm mass, which resulted in the decrease in periodontal health status of the patients after three months of treatment.

The release of biomarkers, as mentioned earlier, is an inflammatory response to the orthodontic tooth movement. Plaque accumulation was evaluated via real time-PCR and tested for the presence for various microbial flora. Young adult group patients ranging from 18 to 25 years were selected for this study as mostly this major group undergoes orthodontic treatment.^32^ In this study, four time-periods were taken, i.e., baseline, 14 days, 1 month and 3 months. The first sample was collected before the bonding or delivery of the aligner. Day 14 was chosen because enzyme turnover time is 7 days and so the forces that were applied to the teeth were still active. The process of indirect resorption starts at 21 days, hence we chose 1 month as the third time period of sample collection. The fourth sample was collected at the third month because leveling is completed in most patients and forces that would produce hyaline zones by tipping the tooth were eliminated.^2^ Fixed appliance patients’ bacterial flora changes from orange to red complex in the first three months, and so in this study time period of three months was taken.^9^^,^^26^^,^^27^ As it has already been mentioned, plaque and gingival indices were used to check the patients’ oral health at first, and the group with a score of 0 was chosen, so that there would be no bias in either group when inflammation or plaque buildup increased over the course of time.

We can infer from this study that there is an increase in the levels of Human ß defensins-2, as well microbial flora, with an increase in the inflammation. Hence, the amount of hBD-2 and microbial flora was maximum at three months and both the parameters were greater in the conventional appliance group. Thus, it may be concluded that conventional orthodontic appliances lead to more inflammation and periodontal issues, as compared to the aligners. However, it must also be focused upon that aligners may not be capable of achieving all the desired tooth movements.^9^ Some orthodontic movements with aligners, like rotation, extrusion and distalization, are difficult to achieve.^33^ Also, the present study was limited to a time period of three months and there may be still some lack of certainty regarding the long-term inflammatory response in both groups.

## LIMITATIONS

This study had a three month follow up, which allows observation of only initial inflammatory changes. Further longer follow up study can be done to strengthen the interpretation.

Additionally, only individuals between the ages of 18 and 25 years, i.e. young adult group having Class I malocclusion, were included in this study. Further studies can be conducted in the future with different age groups, having different malocclusion patients.

## CONCLUSION

- For hBD-2 in GCF, the conventional group showed significantly higher concentration at the first and second months.
- The concentration of the microbes, i.e., *P. gingivalis, P. intermedia, A. actinomycetemcomitans,* and *T. denticola,* was found to be higher in the conventional group than the aligner group at T1, T2 and T3.
- A significantly higher concentration was observed at every time interval in both groups (aligner and conventional) for hBD-2 and different bacteria.
- It is discerned from this study that aligners, as compared to the conventional fixed orthodontic appliance, allows easier and better maintenance of oral hygiene, and thus helps sustaining overall periodontal health status of the patient during the treatment.

## Data Availability

All data generated or analyzed during this study are included in this published article.
